# Clinical and immunopathological spectrum of immunoglobulin M pemphigoid: a multicenter case series

**DOI:** 10.1111/ddg.15838

**Published:** 2025-08-06

**Authors:** Kaan Yilmaz, Stephanie Goletz, Christoph M. Hammers, Katharina Boch, Claudia Zeidler, Henning Wiegmann, Estelle Bergmann, Tina Rastegar Lari, Onur Dikmen, Nina Häring, Robert Strohal, Andreas Kleinheinz, Eva N. Hadaschik, Nina van Beek, Sonja Ständer, Enno Schmidt

**Affiliations:** ^1^ Department of Dermatology University of Lübeck Lübeck Germany; ^2^ Department of Dermatology Heidelberg University Medical Faculty Mannheim Mannheim Germany; ^3^ Lübeck Institute of Experimental Dermatology University of Lübeck Lübeck Germany; ^4^ Department of Dermatology University of Regensburg Regensburg Germany; ^5^ Department of Dermatology University of Kiel Kiel Germany; ^6^ Section Pruritus Medicine and Center for Chronic Pruritus Department of Dermatology University of Münster Münster Germany; ^7^ Charité ‐ Universitätsmedizin corporate member of Freie Universität Berlin and Humboldt‐Universität zu Berlin Institute of Pathology Berlin Germany; ^8^ Department of Dermatology Federal Academic Teaching Hospital Feldkirch Feldkirch Austria; ^9^ Department of Dermatology Clinical Centre Buxtehude Buxtehude Germany; ^10^ Department of Dermatology University of Essen Essen Germany

**Keywords:** autoimmune bullous diseases, IgM, immunobullous diseases, pemphigoid

## Abstract

**Background and Objectives:**

Pemphigoid diseases are primarily mediated by IgG or IgA autoantibodies against the cutaneous basement membrane zone (BMZ). Although recent observations suggest the existence of exclusively IgM‐mediated pemphigoid disease, a larger study is lacking.

**Patients and Methods:**

This prospective multicenter study included ten patients with exclusive IgM deposition along the BMZ by direct immunofluorescence (IF). Circulating IgM was detected by indirect IF and immunoblotting with recombinant BP180.

**Results:**

The cohort, four females and six males with a median age of 77 years, presented predominantly with prurigo‐like lesions without blisters and mucosal involvement. Sera from nine patients demonstrated linear IgM labeling the blister roof of human salt‐split skin by indirect IF. Four patients yielded IgM reactivity against BP180, but none against BP230. In none of the controls (n = 100), anti‐BMZ IgM was seen by direct IF. 1/30 and 3/60 controls with or without pruritic non‐pemphigoid diseases revealed serum IgM reactivity against the BMZ, respectively.

**Conclusions:**

IgM pemphigoid is characterized by exclusive tissue‐bound anti‐BMZ IgM, serum anti‐BMZ IgM reactivity with BP180 as main target antigen, a predominantly non‐bullous clinical phenotype, absence of mucosal involvement, and a rather mild disease course. Our findings indicate that IgM pemphigoid may represent a distinct entity.

## INTRODUCTION

Pemphigoid diseases (PD) define a heterogeneous group of subepidermal autoimmune blistering disorders (AIBD) characterized by autoantibodies against structural proteins of the cutaneous basement membrane zone (BMZ). The three pillars of diagnosis encompass the clinical picture, direct immunofluorescence (IF) microscopy of a perilesional biopsy, and serological tests.^1^, ^2^, ^3^ In PD, direct IF microscopy typically reveals linear depositions of IgG, complement C3, and/or IgA at the BMZ. Tense blisters and erosions on the skin or adjacent mucous membranes represent the clinical manifestation of autoantibody‐induced loss of dermo‐epidermal or epithelial adhesion and are observed in the vast majority of patients.^1^, ^4^

Based on the target antigen, predominant autoantibody isotype, and clinical presentation, six PD entities have been recognized to date: bullous pemphigoid, mucous membrane pemphigoid (MMP), anti‐p200 pemphigoid, pemphigoid gestationis, linear IgA disease (LAD), and epidermolysis bullosa acquisita (EBA). Although the presence of tissue‐bound IgM autoantibodies is routinely examined by direct IF microscopy, the concept of solely IgM‐mediated PD has generated considerable ambiguity over the past decades.^5^ Previous research on the pathogenic role of IgM in PD has been scarce, primarily limited to individual case reports of IgM (bullous) pemphigoid,^6^, ^7^, ^8^, ^9^, ^10^ IgM MMP,^11^, ^12^, ^13^ and IgM EBA.^14^, ^15^, ^16^ More recently, IgM pemphigoid has been highlighted as a potential separate clinical entity with distinct immunopathological features.^17^ So far, the spectrum of clinical presentation, histopathological and immunological aspects as well as treatment responses in IgM pemphigoid have not been prospectively investigated. In the present prospective study, ten PD patients with exclusive anti‐BMZ IgM reactivity by direct IF were characterized.

## MATERIAL AND METHODS

### Patients

This prospective multicenter case study included ten patients from two primary dermatology centers in Lübeck and Hannoversch Münden, Germany, as well as five secondary or tertiary dermatology centers in Germany (Lübeck, Kiel, Buxtehude, Essen) and Feldkirch, Austria. Inclusion criteria were the clinical suspicion of an AIBD which led to a direct IF microscopy of a perilesional skin biopsy and the presence of linear anti‐BMZ autoantibodies exclusively of the IgM isotype by direct IF microscopy. All patients were diagnosed at the routine autoimmune diagnostic laboratory of the University of Lübeck, Germany, from October 2020 to March 2022.

For serological studies, two control groups were included: 60 individuals with clinically suspected AIBD in whom the diagnosis was subsequently excluded by immunopathological methods at the University of Lübeck, and 30 patients with pruritic dermatoses unrelated to AIBD from the University of Münster. All controls were over 70 years of age. Controls with pruritic dermatoses were assessed with regard to to disease entity, etiological classification according to the *International Forum for the Study of Itch* (IFSI),^18^ and intensity of pruritus using the *Worst Itch Numeric Rating Scale* (WI‐NRS) and the *Average Itch Numeric Rating Scale* (AI‐NRS) in the last 24 hours, as described previously.^19^, ^20^ A third control group consisted of 100 consecutive patients aged over 70 years that were investigated by direct IF of a perilesional skin biopsy taken from sun‐exposed skin (arms, legs, or trunk) with suspected AIBD, yet without linear deposits of IgG, IgA and/or C3 at the cutaneous BMZ.

### Immunofluorescence studies

Direct IF microscopy of perilesional skin biopsy specimens was conducted following standard protocols. All biopsies were stained with fluorescein isothiocyanate‐labeled antibodies against human IgG (Dako, California, US), IgA (Euroimmun, Lübeck, Germany), IgM (Euroimmun, Lübeck, Germany), and C3 (MP Biomedicals, California, US).^21^ Indirect IF microscopy on 1 M sodium chloride‐split skin for detection of circulating IgG (Dako, California, US), IgA (Euroimmun Lübeck, Germany), and IgM (Euroimmun, Lübeck, Germany) autoantibodies (diluted 1:2, respectively) was performed as previously reported.^17^ Indirect IF microscopy with primate 1 M NaCl‐split skin, recombinant tetrameric BP180 NC16A, and HEK293 cells transfected with C‐terminal globular domain of BP230 was performed using a BIOCHIP^®^ mosaic (Euroimmun, Lübeck, Germany)^22^ and a FITC‐labelled anti‐IgM antibody (MP Biomedicals, California, USA; diluted 1:50 and 1:100). Sera were diluted 1:10. All IF microscopy specimens were read by experienced physicians specifically trained in this field.

### Immunoblotting

Immunoblotting with *(1)* recombinant BP180 NC16A (aa490‐562), *(2)* recombinant BP180 ectodomain (BP180ec, aa490‐1,497), and *(3)* a recombinant fragment of the C‐terminus of BP180 (BP180(ec)3, aa1,024‐1,270) was performed as described previously.^17^

### Histopathology

Lesional skin biopsy samples were obtained as part of routine diagnosis and examined with hematoxylin‐eosin staining according to standard protocols.

### Ethics Statement

The study adheres to the tenets of the Declaration of Helsinki and was approved by the institutional review board of the University of Lübeck (22‐129, 23–535) and the University of Münster (2023‐487‐b‐S). The patients in this manuscript have given written informed consent to publication of their case details.

## RESULTS

The study included four female and six male patients with a median age of 77 years at diagnosis of IgM pemphigoid, ranging from 60 to 98 years. The median diagnostic interval from patient‐reported first symptom onset to final diagnosis was 12 months and varied between 2 and 26 months (Table 1).

**TABLE 1:** Demographic, clinical, and histopathological features of patients with IgM pemphigoid (n=10).

**Age at diagnosis; years**	
Mean (SD)	78.4 (11.4)
Median (range)	77 (60–98)
**Sex**	**n**
Female	4
Male	6
**Time lag from symptom onset to diagnosis; months**	
Mean (SD)	13 (8.4)
Median (range)	12 (2–26)
**Suspected diagnoses at first presentation**	**n (n=10)**
Chronic prurigo	5
Drug eruption	2
Eczema	1
Parapsoriasis	1
Bullous pemphigoid	1
**Clinical features**	
Itch	10
Erythema/urticarial lesions	9
Excoriated papules/plaques	7
Lichenification	6
Erosions	3
Blisters	2^*^
Pigmentation	1
Mucosal involvement	0
**Histological features**	
Lymphocytic infiltrate	9
Eosinophils	6
Parakeratosis	5
Spongiosis	5
Acanthosis	5
Neutrophils	3
Dermal edema	3
Hypergranulosis	2
Hypogranulosis	1
Subepidermal blistering	1

*Abbr*.: n, number; SD, standard deviation

* Anamnestic data, no blisters were detected at first presentation or during follow‐up.

### Clinical presentation

**FIGURE 1:**
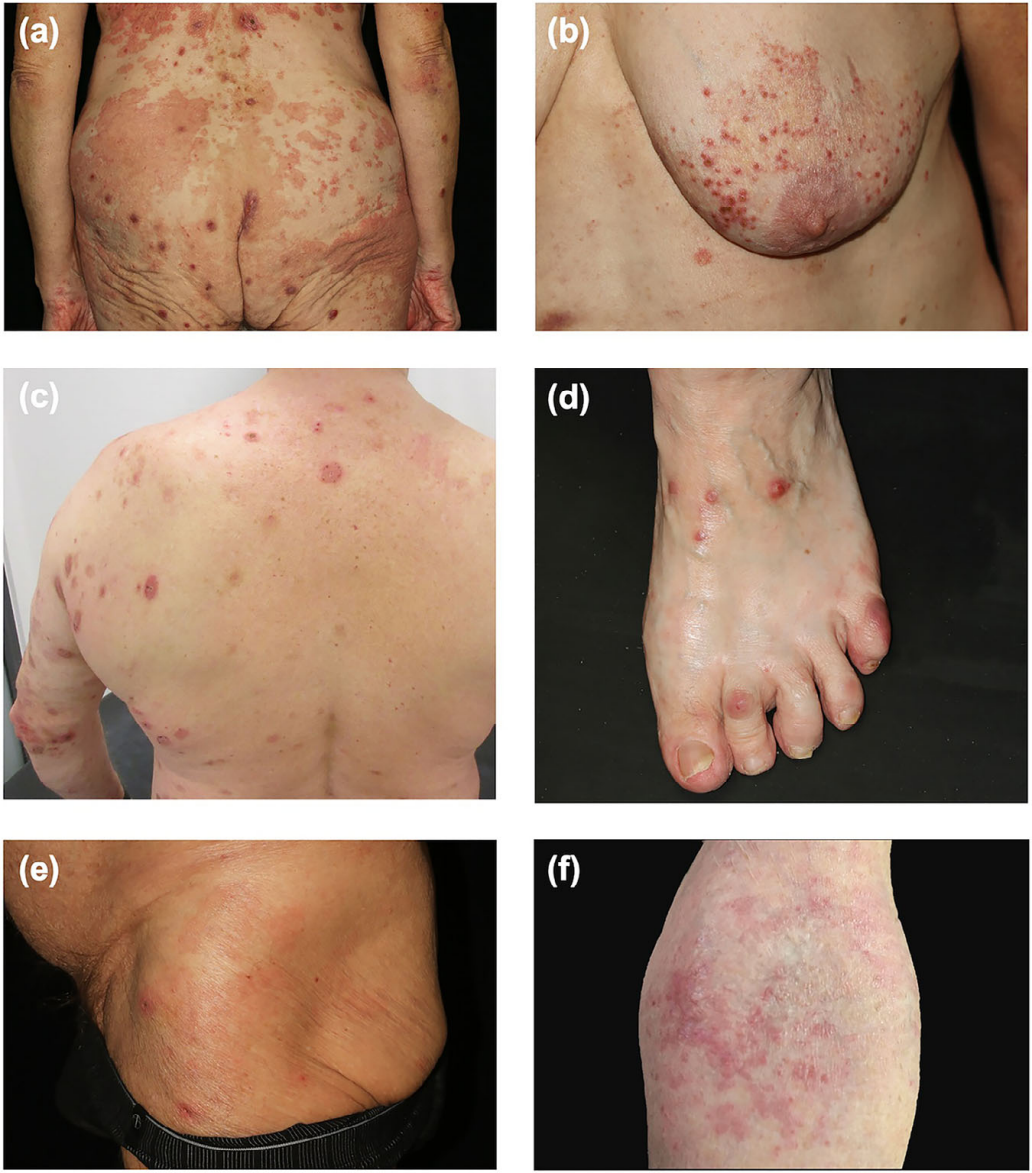
Clinical manifestations of IgM pemphigoid. (a) Confluent, erythematous, well‐defined urticarial plaques along with multiple excoriated papules on the buttocks and lower back in patient 6. (b) Multiple grouped pinhead‐sized excoriated papules with an erythematous base on the breast in patient 6. (c) Disseminated discoid erythematous plaques on the upper back and left arm in patient 5. (d) Erythematous papules and plaques on the dorsum of the left foot in patient 6. (e) Erythematous macules and plaques with few excoriated papules on the left hip in patient 8. (f) Erythematous macules on the antecubital fossa in patient 10.

All patients exhibited skin disease accompanied by itch. Erythema and urticarial lesions were the most prevalent skin manifestations that occurred in 90% of the patients, followed by excoriated papules and plaques (in 70%), and lichenification (in 60%). Erosions were observed in three patients (30%), whereas one patient (10%) showed hyperpigmentation. Two patients (20%) reported a history of skin blisters; however, no blisters were observed at initial presentation or during follow‐up visits. No mucosal lesions were detected in any patient, including oral, nasal, ocular, or anogenital involvement (Table 1, Figure 1).

In all patients, direct IF was performed to exclude PD. The clinical diagnoses were chronic prurigo in five patients, drug eruption to hydrochlorothiazide and lamotrigine, respectively in two patients, and eczema, parapsoriasis, and bullous pemphigoid in one patient each (Table 1, Table S1).

### Histopathological features

**FIGURE 2:**
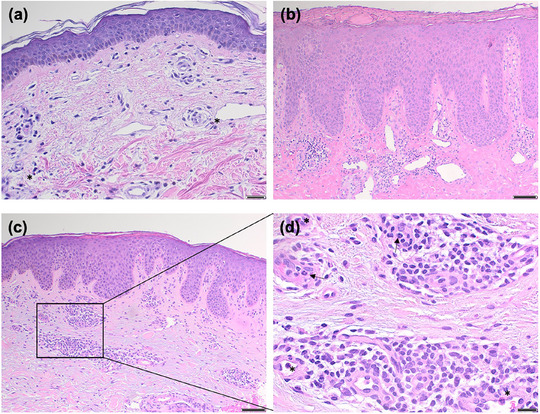
Histopathological spectrum of IgM pemphigoid. (a) Superficial perivascular dermatitis with mild dermal edema and mild lymphohistiocytic infiltrates with scattered eosinophils (asterix) in patient 3 (hematoxylin‐eosin staining [HE], scale bar: 100 µm). (b) Chronic nodular prurigo‐like parakeratosis, irregular acanthosis, and hypergranulosis in patient 6. In the upper dermis, fibrosis and patchy lymphohistiocytic infiltrates are present. Scale bar, 100 µm. (c) Mild spongiotic and psoriasiform dermatitis in patient 10 (HE, scale bar: 100 µm). (d) Perivascular lymphocytic infiltrates with admixed neutrophils (arrows) and few eosinophils (asterix) at higher magnification of (c) (scale bar: 20 µm).

Epidermal alterations were frequently observed in lesional skin samples, including parakeratosis (in 5 patients), acanthosis (in 5 patients), spongiosis (in 5 patients), hypergranulosis (in 2 patients), and hypogranulosis (in 1 patient). Subepidermal blistering was detected in 1 patient. Edema of the superficial dermis, a sign of urticarial dermatitis, was found in three cases. Dermal infiltrates mostly included lymphocytes (in 9 patients), with admixed eosinophils (in 6 patients) and neutrophils (in 3 patients) (Table 1, Figure 2).

### Immunopathological characteristics

**FIGURE 3:**
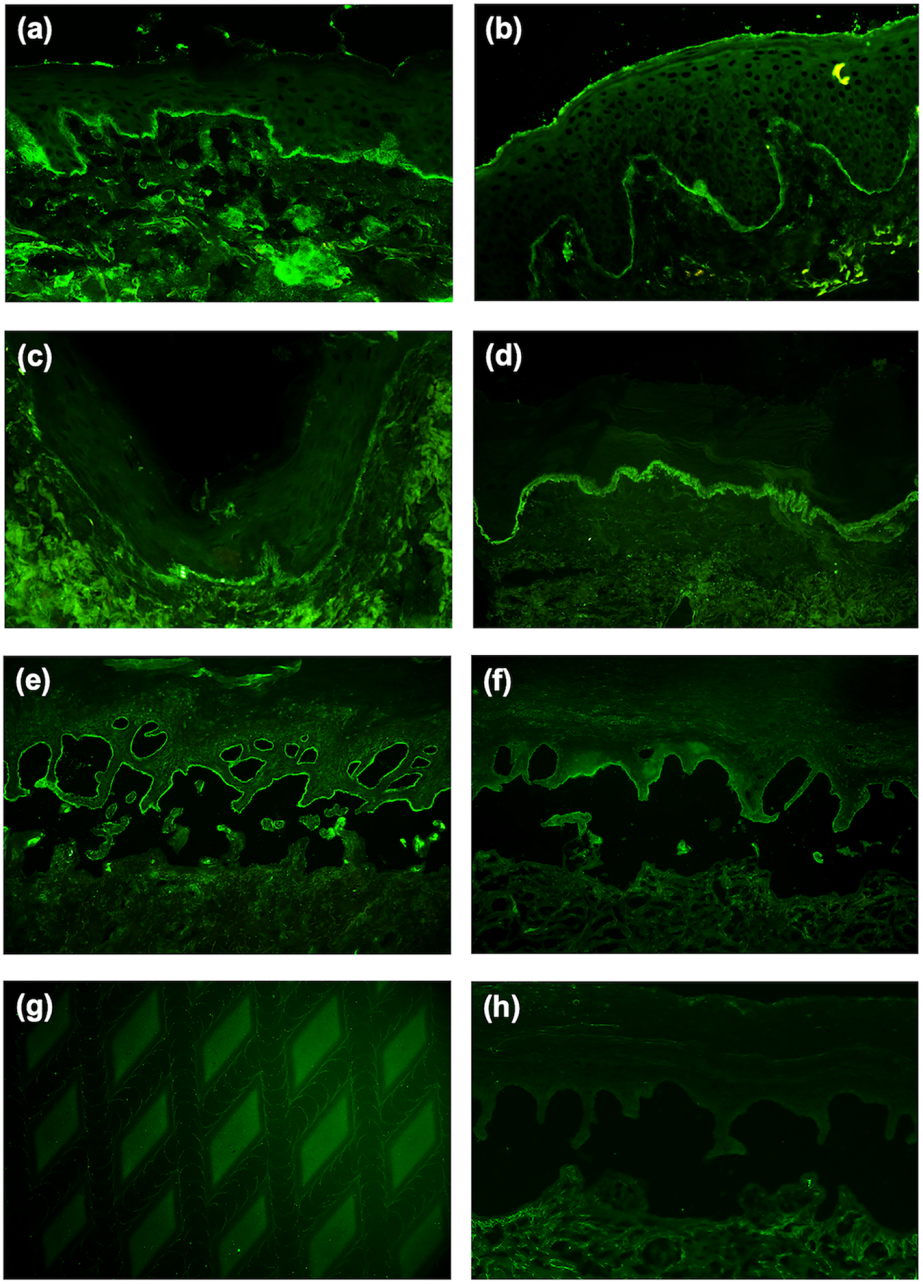
Immunopathological features of IgM pemphigoid. (a–c) Direct immunofluorescence (IF) microscopy of a perilesional skin biopsy with sharply delineated, distinct, linear depositions of IgM at the cutaneous BMZ in (a) patient 9, (b) patient 10 and (c) in patient 3. (d) Thick, homogenous band of IgM deposits at the basement membrane zone in a patient with lupus erythematosus in comparison. (e) Indirect IF microscopy on human 1 M salt‐split skin with IgM deposits labeling the epidermal side of the artificial split in patient 6. (f) Linear IgM deposition along the blister roof of salt‐split skin by multiplex IF BIOCHIP^®^ mosaic (Euroimmun, Lübeck, Germany) in patient 4. (g) IgM autoantibodies against BP180 NC16A by BIOCHIP^®^ mosaic with recombinant BP180 in patient 3. (h) No IgM deposition by indirect IF microscopy on salt‐split skin was seen in a control patient with atopic dermatitis.

Direct IF microscopy of all perilesional skin biopsies showed exclusive labeling of the cutaneous BMZ by IgM, in the absence of IgG, IgA, and complement C3 (Figure 3a–c). In nine cases, the serration pattern could not be determined; in one biopsy, an n‐serrated pattern was seen (Table 2, Table S2). In 100 biopsies from sun‐exposed skin of consecutive patients > 70 years of age with suspected AIBD, yet without deposits of IgG, IgA and C3, linear IgM deposition at the BMZ was found in none of those. In these controls, cytoid bodies and granular IgM deposits were observed in 3% and in 1%, respectively (Table S3).

**TABLE 2:** Immunopathological characteristics of patients with IgM pemphigoid (n=10).

	n
**Linear deposition of IgM at the cutaneous BMZ by direct IF**	
n‐serrated pattern	1
u‐serrated pattern	0
Undetermined pattern	9
Additional IgG/IgA reactivity	0
**Indirect IF on human salt‐split skin**	
IgM along epidermal side	9
IgM along dermal side	0
Negative	1
Additional IgG/IgA reactivity	0
**Target antigen**	
BP180 NC16A (IgM)	3
BP180 ectodomain (IgM)	2
BP180(ec)3 (C‐terminal, IgM)	0
BP230 (IgM)	0
Unknown	6
BP180 NC16A (IgG)	1^*^

*Abbr*.: n, number; BMZ, basement membrane zone; IF, immunofluorescence; ec, ectodomain

* Additional IgG reactivity against BP180 NC16A by ELISA (27 U/ml; normal, < 20 U/ml) in patient 3.

**FIGURE 4:**
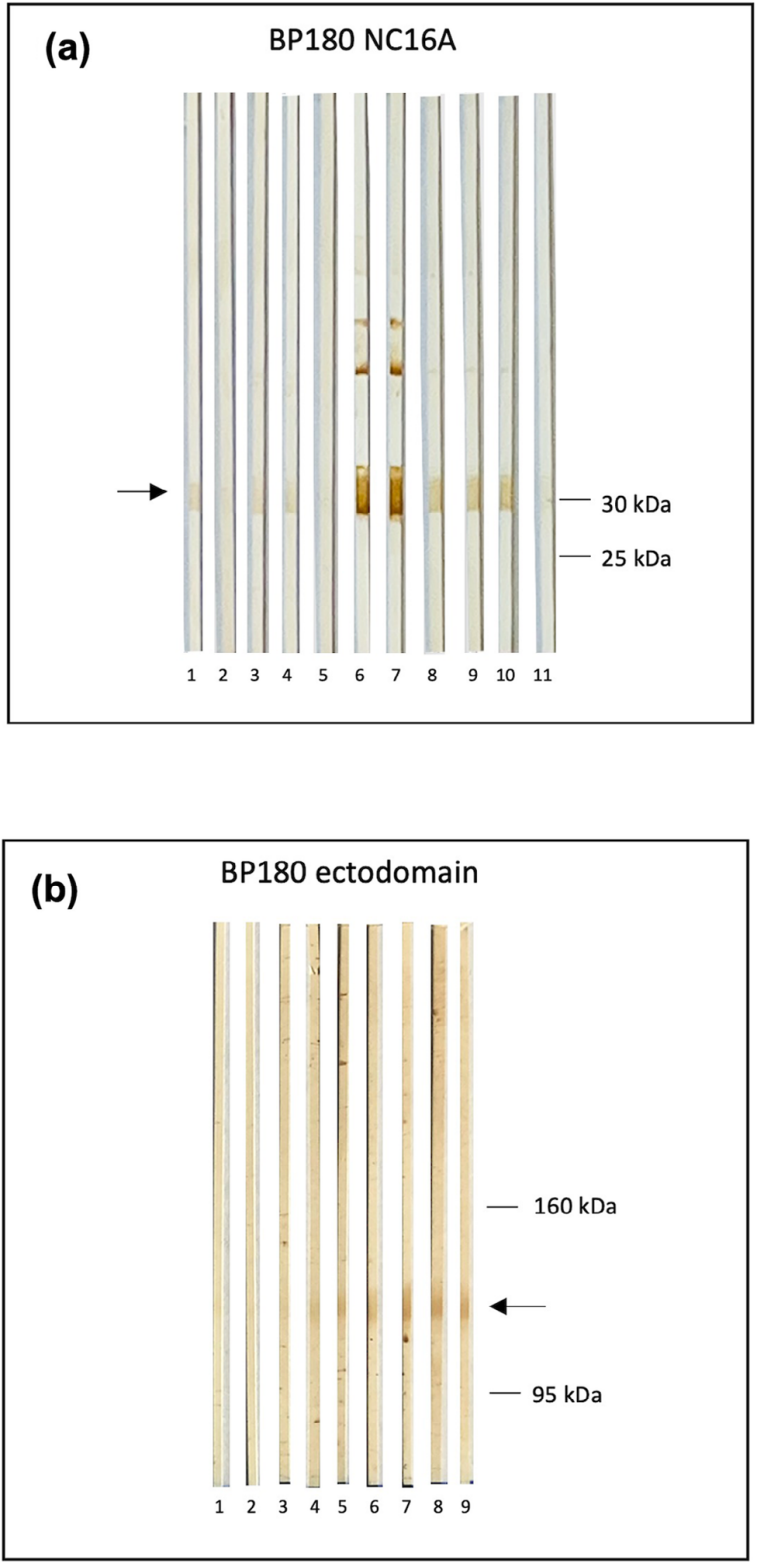
Immunoblot analyses of IgM pemphigoid. (a) Immunoblot analyses with recombinant BP180 NC16A. Serum IgM reactivity was detected in patient 3 (lanes 9 and 10), but not in patient 4 (lane 11). Sera of healthy blood donors served as negative controls (lanes 1–5). Sera of three previously reported patients with IgM pemphigoid^17^ were used as positive controls (lanes 6–8). (b) By immunoblotting with the recombinant BP180 ectodomain, patient 6 and patient 7 displayed serum IgM reactivity (lanes 8 and 9, respectively). Normal human sera are shown in lanes 1–5, and two previously reported patients with IgM pemphigoid^17^ in lanes 6 and 7. Migration positions of the recombinant proteins are indicated by arrows, molecular weight markers in kDa are shown at the right.

In nine of the ten patients with IgM pemphigoid, circulating IgM autoantibodies were detected by indirect IF microscopy on human salt‐split skin, all of which bound to the epidermal side of the artificial blister (Figure 3e). Immunoblotting with the recombinant NC16A domain of BP180 (Figure 4a) and the recombinant BP180 ectodomain (Figure 4b) showed IgM reactivity in three and two patients, respectively. One patient's serum was reactive in both immunoblot studies (patient 6, Table S2). Immunoblotting with a C‐terminal stretch of BP180 (BP180(ec)3) was negative for IgM autoantibodies in all patients. Circulating IgG or IgA autoantibodies were not found by indirect IF microscopy in any of the patients, while 1/10 patients (patient 3) exhibited additional low IgG reactivity against BP180 NC16A by enzyme‐linked immunosorbent assay (ELISA; 27 U/ml; normal, < 20 U/ml) (Table 2).

Using the BIOCHIP® mosaic, no anti‐BP230 IgM autoantibodies were detected. In contrast, IgM reactivity at the epidermal side of salt‐split skin was confirmed in seven of nine patients, and anti‐BP180 NC16A IgM reactivity was observed in one of three sera that reacted with this protein by immunoblotting (Table S2, Figure 3f–g). In four patients, sera were available during the disease course. In none of them, Ig subtype switching was detected (data not shown).

In serological control groups, circulating IgM autoantibodies were detected by indirect IF microscopy on salt‐split skin in 1/30 patients (3.33%) with well‐characterized pruritic non‐PD dermatoses and in 3/60 patients (5%) with clinically suspected, yet immunopathologically excluded PD (Figure 3h; Tables S4, S5).

### Treatment responses

The disease course was monitored in eight patients for a median period of 10 months (range, 4–28 months). Two patients were lost to follow‐up. Disease‐specific therapeutic outcomes were measured as proposed by an international panel of experts^23^ (Table S1).

Topical potent and super‐potent corticosteroids were used in all patients, of which four patients received additional systemic therapy including prednisolone, azathioprine, mycophenolate mofetil (MMF), doxycycline, dapsone, and rituximab. Among the patients receiving topical therapy only (n = 5), three achieved complete remission off therapy, while the remaining two reached partial remission on minimal therapy. Of the three patients who received additional systemic treatment, two achieved partial remission on minimal therapy during systemic monotherapy with dapsone and azathioprine, respectively. One patient experienced a relapse despite sequential treatment with doxycycline, azathioprine, and mycophenolate mofetil (MMF) in combination with oral prednisolone. In this case, disease control was achieved only after the addition of rituximab. (patient 5, Table S1).

## DISCUSSION

IgM‐mediated PD has only recently been proposed as a distinct entity. Early studies described the clinical heterogeneity of affected patients but questioned the pathogenic relevance of IgM autoantibodies targeting the cutaneous BMZ.^5^ Accordingly, no target antigen has been identified for IgM pemphigoid, and the IgM deposits were interpreted as a nonspecific hypersensitivity response, similar to what is observed in conditions such as urticaria, leukocytoclastic vasculitis, or hypersensitivity dermatitis.^7^ Interestingly, only one of the 25 patients evaluated in the early study by Velthuis et al. was in fact considered to have bullous pemphigoid with cutaneous blisters, IgM and complement C3 depositions along the BMZ by direct IF microscopy, and circulating anti‐BMZ autoantibodies.^5^ Besides this single case, early reports failed to sufficiently define IgM serum autoantibodies.^24^ Subsequently, the term “linear IgM dermatosis of pregnancy” was introduced, referring to a clinical phenotype with transient pruritic, erythematous papules and urticarial lesions usually occurring in the third trimester, accompanied by linear IgM depositions at the cutaneous BMZ.^25^, ^26^ Although the proposal of a novel gestational entity has not met general acceptance, circulating IgM autoantibodies targeting BP180 and BP230 were found in 10–14% of pregnant women, indicating a potential link between pregnancy and low degree of cutaneous IgM autoreactivity.^27^ In our cohort, no patient was at reproductive age and the age structure of our patients resembled that of bullous pemphigoid which characteristically arises in the late 70s.^1^ Notably, between October 2020 to March 2022, 527 patients were diagnosed with PD at the autoimmune diagnostic laboratory of the Department of Dermatology, University of Lübeck, Germany, including only ten cases of IgM pemphigoid (about 1.9% of all PD cases).

Some PD cases with IgM anti‐BMZ reactivity have previously been associated with Waldenström macroglobulinemia.^28^, ^29^ In contrast, in none of our patients, Waldenström macroglobulinemia was evident. Despite several attempts, the antigenic target of IgM autoantibodies in PD remained enigmatic, except in a single case of IgM EBA with anti‐type VII collagen IgM.^14^ More recently, circulating IgM autoantibodies against BP180 in all three patients with IgM pemphigoid were demonstrated.^17^ Concordantly, BP180 was detected as autoantigen in a patient with IgM pemphigoid using super‐resolution imaging, suggesting that the C‐terminus of BP180 might be a target antigen.^9^

In the present study, circulating anti‐BMZ IgM autoantibodies were detected in nine of ten patients, while four of ten exhibited IgM autoantibodies targeting BP180. The extracellular portion of the 16th non‐collagenous (NC16A) domain—the immunodominant region of BP180 in bullous pemphigoid—was the most frequently targeted autoantigen (30%), followed by the BP180 ectodomain. No target antigen could be identified in the other six patients, while IgM labeled the roof of the artificial blister by indirect IF microscopy in all these cases. This may be explained by lower levels of IgM autoantibodies in these patients and by the fact that both immunoblotting and multiplex indirect immunofluorescence assays were not optimized for the detection of IgM reactivity. Alternatively, IgM autoantibodies may target other epidermal components of the BMZ, such as BP230, α6β4 integrin, or plectin; however, their detection may be limited by the lack of validated assays for identifying IgM autoreactivity against these potential antigens. Interestingly, a serration pattern of linear IgM deposits by direct IF microscopy was identified in only one patient, which is notably lower than the detection frequency in other PD, where a serration pattern can be observed in about 75% of cases.^30^ This discrepancy may be attributed to the structural properties of IgM, which predominantly exists in a pentameric form.^31^ This configuration may impede the formation or detection of a distinct serration pattern.

To sufficiently address the controversy surrounding the diagnostic specificity of linear IgM deposition, the present study opted to include several control groups. First, samples obtained from sun‐exposed areas from 100 individuals without AIBD did not reveal linear anti‐BMZ IgM binding by direct IF microscopy. Of note, cytoid bodies and granular IgM deposits were identified in 3% and 1% of control cases, respectively (Table S3). IgM‐positive cytoid‐bodies, particularly when multiple and arranged in clusters, can be found in conditions such as lupus erythematosus and lichen planus.^32^ Conversely, sparse cytoid bodies may be encountered across a broad spectrum of chronic inflammatory dermatoses, including cutaneous graft‐versus‐host disease, lupus erythematosus, erythema multiforme, leukocytoclastic vasculitis, elastosis perforans serpiginosa, and sun‐exposed skin of healthy individuals.^33^, ^34^, ^35^ In a comprehensive analysis of over 1,000 cutaneous specimens by direct IF microscopy, cytoid bodies were present in 10.8% of cases, with the IgM isotype accounting for 72.6%.^33^ The same study demonstrated that granular IgM deposition may also be observed in chronic inflammatory dermatoses including lupus erythematosus.^33^ Second, immunoserological studies were conducted using sera from patients with well‐characterized chronic pruritus disorders as well as from individuals with clinically suspected, but immunopathologically excluded, PD. Congruently, IgM depositions by indirect IF microscopy on salt‐split skin were observed in only few cases (1/30 and 3/60, respectively). IgM reactivity against BP180 NC16A was detected by immunoblotting in 6.3 % of the sera (Tables S4, S5), which may be explained by the lack of assay optimization for IgM detection. Collectively, our findings indicate that IgM pemphigoid can be diagnosed based on the clinical presentation, direct IF microscopy, and indirect IF microscopy using salt‐split skin.

By direct IF microscopy, it is important to differentiate linear anti‐BMZ reactivity typical and diagnostic for PD from band‐like IgM deposits at the cutaneous BMZ as seen in LE, and possibly in some patients with other inflammatory dermatoses or sun‐exposed skin. Similar to other PD, IgM pemphigoid is characterized by sharply demarcated, thin, and continuous linear IgM deposits along the BMZ (Figure 3a–c). In contrast, lupus erythematosus typically presents with broad, band‐like, homogeneous or granular IgM deposits (Figure 3d), a pattern attributed to immune complex deposition or antigen release or modification via UV‐induced keratinocyte damage.^36^, ^37^ Notably, similar IgM deposits may also be observed in sun‐exposed skin, other chronic inflammatory dermatoses, and even healthy individuals. This deposition pattern is often granular or band‐like, albeit less intense, frequently focal or interrupted, and is generally considered non‐specific.^36^, ^38^

The absence of mucosal involvement in our patients is in line with previous reports on IgM PD strictly limited to the skin^9^, ^39^ or with only mild oral lesions.^6^ Similarly, all hitherto reported patients with IgM EBA lacked lesions at mucosal sites.^14^, ^15^, ^16^ In contrast, mucosal surfaces are affected in 10–20% in bullous pemphigoid^40^ and approximately half of the patients with EBA.^41^ In fact, only few patients with IgM MMP have been reported so far, varying from minimal conjunctival lesions to severely scarring, debilitating ocular disease.^11^, ^12^, ^13^

Recent evidence indicates that in IgM pemphigoid, circulating autoantibodies can internalize BP180 *ex vivo*, albeit without complement activating or blister inducing capacity.^17^ This concurs well with our findings that no patient revealed complement C3 depositions at the BMZ by direct IF microscopy. Despite its well‐known complement‐fixing properties, IgM activates the complement system only upon binding to surface antigens.^42^ Moreover, hexameric IgM, a far more potent complement activator than IgM pentamer, only accounts for less than 5% of total IgM.^31^, ^43^ It can thus be hypothesized that low‐affinity interactions between predominantly pentameric IgM and BP180 may lead to insufficient complement activation, which, in turn, results in decreased inflammatory response in the skin, and the absence of histological and clinical blistering. Accordingly, in previous reports of IgM pemphigoid with bullous phenotype, linear deposits of complement C3 at the BMZ were consistently detected alongside IgM autoantibodies,^6^ whereas the presence of skin‐bound C3c did not necessarily result in clinical blisters.^9^, ^17^ Therefore, it seems tempting to infer that IgM pemphigoid might be exacerbated or undergo phenotypical shifts by further contribution of complement C3 and possibly IgG and/or IgA autoantibodies. It needs to be established in further studies whether the diagnosis of IgM pemphigoid requires the lack of additional IgG, IgA, and C3 reactivity by direct IF microscopy like in the present study or, in line with LAD, also includes patients with weak IgG, IgA, and/or C3 deposits. In this context, it may be advisable to repeat direct IF microscopy of a perilesional skin biopsy during the course of disease, particularly in patients with new skin lesions and changes in the clinical presentation.

Most of our patients presented with pruritic papules and plaques, erythema, and lichenification in the absence of blisters, reinforcing most recent findings.^17^ Only two patients had blisters prior to their first consultation, while no blistering was observed at the initial presentation or during follow‐up visits. In this regard, IgM pemphigoid seems to differ from classical bullous pemphigoid, although in latter disease about 20% of patients present with non‐bullous variants resembling chronic prurigo, eczema, intertrigo, ecthyma gangrenosum, erythroderma, and toxic epidermal necrolysis.^44^

Of note, the mean diagnostic delay in our study (12 months) was almost twice as high as that in bullous pemphigoid (6.1 months).^44^ This may be due to the rather unspecific and mild clinical picture of IgM pemphigoid that is also reflected by the mild disease course with half of our patients achieving complete remission off therapy or partial remission on minimal therapy within few months when treated with topical corticosteroids alone. This confirms recent findings indicating a mild disease course in IgM pemphigoid.^17^

Taken together, the present study describes IgM pemphigoid as a distinct and potentially underdiagnosed clinical entity characterized by *(1)* exclusive anti‐BMZ IgM autoantibodies by direct IF microscopy, *(2)* serum IgM reactivity against the epidermal side of salt‐split skin, *(3)* BP180 as main target antigen, *(4)* a predominantly non‐bullous phenotype in the absence of mucosal involvement, and *(5)* a rather mild disease course.

## FUNDING

This work received infrastructural support through the DFG by the Collaborative Research Center CRC 1526 *Pathomechanisms of Antibody‐mediated Autoimmunity (PANTAU)*, *grant no. 454193335*, the Schleswig‐Holstein Excellence Cluster *Precision Medicine in Chronic Inflammation* (DFG EXC 2167/1), and funding from the University of Lübeck (CS06‐2019 to CMH).

## CONFLICT OF INTEREST STATEMENT

E.S. has a scientific cooperation and patents with Euroimmun, Lübeck, Germany. S.G., C.M.H., and N.v.B. hold a patent with Euroimmun, Lübeck, Germany. All other authors declare no conflict of interest.

## Supporting information



Supplementary information
