# Supplementary material for: Clinical and immunopathological spectrum of immunoglobulin M pemphigoid: a multicenter case series
Source: J Dtsch Dermatol Ges. 2025 Aug 6;23(12):1555–64. doi: 10.1111/ddg.15838 (PMC12697332; doi:10.1111/ddg.15838)
Supplement: Supplementary file 1 — Supplementary information [file DDG-23-1555-s001.docx]

[[Online supplement]]

TABLE S1 Detailed clinical characteristics of patients with IgM pemphigoid.

| **Case #** | **Age** | **Sex** | **Time lag to diagnosis** | **Initially suspected diagnosis** | **Clinical picture** | | | | | | | | **Therapeutic regimen** | | **Follow-up (months)** | **Outcome** |
| --- | --- | --- | --- | --- | --- | --- | --- | --- | --- | --- | --- | --- | --- | --- | --- | --- |
|  |  |  |  |  | ***Erythema/ urticarial lesions*** | ***Erosions*** | ***Blisters*** | ***Excoriated papules/ plaques*** | ***Lichenification*** | ***Itch*** | ***Pigmentation*** | ***Mucosal affection*** | ***Topical CS*** | ***Systemic*** |  |  |
| 1 | 98 | m | Several months | Chronic prurigo | + | + | – | + | + | + | – | – | + | – | 4 | PRMT |
| 2 | 92 | f | 9 months | Eczema | + | + | +* | + | + | + | – | – | + | – | 13 | CROT |
| 3 | 75 | f | 26 months | Chronic prurigo | + | – | – | + | – | + | – | – | + | Dapsone | 28 | PRMT |
| 4 | 79 | m | 13 months | Bullous pemphigoid | + | + | – | + | – | + | – | – | + | – | 17 | PRMT |
| 5 | 60 | m | 4 months | Chronic prurigo | + | – | – | + | + | + | + | – | + | *Initial*: doxycycline, azathioprine  *Current*: MMF, prednisolone, rituximab | 10 | CDA |
| 6 | 69 | f | 26 months | Chronic nodular prurigo | + | – | – | + | – | + | – | – | + | Prednisolone | N.d. | LTF |
| 7 | 70 | m | 2 months | HCT-associated drug eruption | + | – | +* | – | – | + | – | – | + | – | 5 | CROT |
| 8 | 84 | m | 12 months | Parapsoriasis | + | – | – | – | + | + | – | – | + | Azathioprine^#^ | 8 | PRMT |
| 9 | 73 | m | 13 months | Chronic prurigo | – | – | – | + | + | + | – | – | + | – | 10 | CROT |
| 10 | 84 | f | 12 months | Drug eruption secondary to lamotrigine | + | – | – | – | + | + | – | – | + | – | N.d. | LTF |

*Abbr.:* CDA, control of disease activity; CROT, complete remission off therapy; CS, corticosteroids; f, female; HCT, hydrochlorothiazide; m, male; LTF, lost to follow-up, MMF, mycophenolate mofetil; n.d., not done; PRMT, partial remission on minimal therapy

*Anamnestic data, no blisters were detected at first presentation or during follow-up, ^#^initiated to treat a non-dermatological condition^,^ but improved the skin disease as well.

TABLE S2 Detailed histological and immunopathological features of patients with IgM pemphigoid.

| **Case #** | **Direct IF BMZ** | **Indirect IF on salt-split skin** | **Immunoblotting** | | | **Biochip** | | | **Histopathological features** | | | | | | | | | |
| --- | --- | --- | --- | --- | --- | --- | --- | --- | --- | --- | --- | --- | --- | --- | --- | --- | --- | --- |
|  |  |  | ***BP180 NC16A IgM*** | ***BP180ec***  ***IgM*** | ***BP180(ec)3 (C-terminal)***  ***IgM*** | ***Indirect IF on salt-split skin*** | ***BP180 NC16A IgM*** | ***BP230 IgM*** | ***Parakeratosis*** | ***Hypergranulosis*** | ***Hypogranulosis*** | ***Acanthosis*** | ***Spongiosis*** | ***Neutrophils*** | ***Eosinophils*** | ***Lymphocytic infiltrate*** | ***Subepidermal blistering*** | ***Dermal edema*** |
| 1 | IgM (und.) | negative | + | – | – | Negative | – | – | + | – | – | + | – | + | – | + | + | – |
| 2 | IgM (und.) | IgM  epidermal side | – | – | – | IgM epidermal side | – | – | + | + | – | – | + | – | + | + | – | + |
| 3 | IgM (und.) | IgM  epidermal side | + | – | – | IgM epidermal side | + | – | – | – | – | – | – | – | + | + | – | – |
| 4 | IgM  (und.) | IgM  epidermal side | – | – | – | IgM epidermal side | – | – | – | – | – | + | + | + | + | – | – | – |
| 5 | IgM  (und.) | IgM  epidermal side | – | – | – | IgM epidermal side | – | – | – | – | – | + | – | – | – | + | – | – |
| 6 | IgM (und.) | IgM  epidermal side | + | + | – | Negative | – | – | + | + | – | + | – | – | – | + | – | – |
| 7 | IgM (und.) | IgM  epidermal side | – | + | – | IgM epidermal side | – | – | – | – | – | – | + | – | + | + | – | + |
| 8 | IgM (und.) | IgM  epidermal side | – | – | – | Negative | – | – | + | – | – | – | + | – | – | + | – | – |
| 9 | IgM  (n-serrated) | IgM  epidermal side | – | – | – | IgM epidermal side | – | – | – | – | – | – | – | – | + | + | – | + |
| 10 | IgM (und.) | IgM  epidermal side | – | – | – | IgM epidermal side | – | – | + | – | + | + | + | + | + | + | – | – |

*Abbr.:* BMZ, basement membrane zone; ec, ectodomain; IB, immunoblot; IF, immunofluorescence; und., undetermined serration pattern

TABLE S3 Direct IF microscopy analysis of IgM reactivity in consecutive samples of patients > 70 years of age with suspected AIBD, yet without linear IgG, IgA, and C3 deposits at the cutaneous BMZ. Biopsies stem from potentially sun-exposed areas.

| **Case #** | **Age** | **Sex** | **IgM deposits in direct IF microscopy** | | |
| --- | --- | --- | --- | --- | --- |
|  |  |  | *Arm* | *Leg* | *Trunk* |
| 1 | 82 | F |  |  | Neg. |
| 2 | 80 | F |  |  | Neg. |
| 3 | 76 | F |  | Neg. |  |
| 4 | 85 | M |  | Neg. |  |
| 5 | 87 | F |  | Neg. |  |
| 6 | 89 | F |  |  | Neg. |
| 7 | 77 | F |  |  | Neg. |
| 8 | 86 | F |  |  | Neg. |
| 9 | 85 | M | Neg. |  |  |
| 10 | 85 | M |  | Neg. |  |
| 11 | 84 | M | Neg. |  |  |
| 12 | 81 | F |  |  | Neg. |
| 13 | 90 | M |  |  | Neg. |
| 14 | 78 | F |  |  | Neg. |
| 15 | 81 | M |  |  | Neg. |
| 16 | 76 | F |  | Neg. |  |
| 17 | 84 | M | Neg. |  |  |
| 18 | 86 | M |  |  | Neg. |
| 19 | 86 | F |  |  | Neg. |
| 20 | 85 | M |  | Neg. |  |
| 21 | 82 | F |  | Neg. |  |
| 22 | 83 | M |  | Neg. |  |
| 23 | 79 | M |  |  | Neg. |
| 24 | 83 | F |  | Neg. |  |
| 25 | 75 | F | Neg. |  |  |
| 26 | 86 | M |  | Neg. |  |
| 27 | 88 | M |  | Neg. |  |
| 28 | 91 | M |  |  | Cyt. bod. |
| 29 | 88 | F |  |  | Neg. |
| 30 | 75 | F |  |  | Neg. |
| 31 | 88 | F |  | Neg. |  |
| 32 | 89 | F |  | Neg. |  |
| 33 | 79 | M |  |  | Neg. |
| 34 | 87 | F |  |  | Neg. |
| 35 | 84 | F | Neg. |  |  |
| 36 | 83 | M |  |  | Neg. |
| 37 | 86 | F |  | Neg. |  |
| 38 | 81 | F | Neg. |  |  |
| 39 | 84 | F |  | Neg. |  |
| 40 | 86 | F |  | Neg. |  |
| 41 | 83 | M |  |  | Neg. |
| 42 | 83 | F |  | Neg. |  |
| 43 | 90 | M |  | Neg. |  |
| 44 | 80 | M |  |  | Neg. |
| 45 | 88 | M |  | Neg. |  |
| 46 | 84 | F |  | Neg. |  |
| 47 | 85 | F | Neg. |  |  |
| 48 | 81 | F |  | Neg. |  |
| 49 | 86 | F |  | Neg. |  |
| 50 | 86 | F | Gran. |  |  |
| 51 | 80 | M | Neg. |  |  |
| 52 | 76 | F | Neg. |  |  |
| 53 | 85 | F |  | Neg. |  |
| 54 | 78 | M |  | Neg. |  |
| 55 | 91 | M |  |  | Neg. |
| 56 | 77 | F |  | Neg. |  |
| 57 | 91 | M |  | Neg. |  |
| 58 | 88 | F |  | Neg. |  |
| 59 | 87 | F |  |  | Neg. |
| 60 | 82 | F |  |  | Neg. |
| 61 | 93 | F | Neg. |  |  |
| 62 | 81 | F | Neg. |  |  |
| 63 | 89 | F | Neg. |  |  |
| 64 | 75 | M |  | Neg. |  |
| 65 | 87 | F | Neg. |  |  |
| 66 | 76 | F | Neg. |  |  |
| 67 | 86 | M |  | Neg. |  |
| 68 | 80 | F | Neg. |  |  |
| 69 | 76 | M |  |  | Neg. |
| 70 | 85 | F |  | Neg. |  |
| 71 | 86 | F |  |  | Neg. |
| 72 | 79 | M |  | Neg. |  |
| 73 | 80 | F |  | Cyt. bod. |  |
| 74 | 88 | M |  |  | Neg. |
| 75 | 80 | M |  | Cyt. bod. |  |
| 76 | 84 | M |  | Neg. |  |
| 77 | 78 | M | Neg. |  |  |
| 78 | 82 | F |  | Neg. |  |
| 79 | 87 | F |  | Neg. |  |
| 80 | 90 | F | Neg. |  |  |
| 81 | 94 | M |  |  | Neg. |
| 82 | 85 | M |  | Neg. |  |
| 83 | 79 | M |  |  | Neg. |
| 84 | 85 | M | Neg. |  |  |
| 85 | 81 | M |  | Neg. |  |
| 86 | 83 | M |  | Neg. |  |
| 87 | 84 | M |  | Neg. |  |
| 88 | 79 | M |  |  | Neg. |
| 89 | 82 | F |  |  | Neg. |
| 90 | 85 | M |  | Neg. |  |
| 91 | 82 | M |  |  | Neg. |
| 92 | 88 | M |  | Neg. |  |
| 93 | 78 | F |  | Neg. |  |
| 94 | 98 | M |  | Neg. |  |
| 95 | 86 | M |  |  | Neg. |
| 96 | 86 | F |  | Neg. |  |
| 97 | 83 | F |  |  | Neg. |
| 98 | 75 | M |  | Neg. |  |
| 99 | 86 | M |  |  | Neg. |
| 100 | 86 | F |  | Neg. |  |

*Abbr.:* Cyt. bod., cytoid bodies; F, female; Gran., granular; IF, immunofluorescence; M, male; Neg., negative

TABLE S4 Immunoserological findings in patients > 70 years of age with pruritic dermatoses other than AIBD.

| **Case #** | **Age** | **Sex** | **Diagnosis** | **IFSI Group** | **Age at diagnosis** | **Intensity of Pruritus** | | **Indirect IF on**  **salt-split skin (IgM)** | **Immunoblotting with BP180 NC16A (IgM)** |
| --- | --- | --- | --- | --- | --- | --- | --- | --- | --- |
|  |  |  |  |  |  | *WI-NRS/ 24h* | *AI-NRS/ 24h* |  |  |
| 1 | 83 | F | Drug eruption | 1 | 77 | 10 | 8 | - | - |
| 2 | 81 | M | Atopic dermatitis | 1 | 78 | 7 | 5 | - | - |
| 3 | 85 | M | Chronic nodular prurigo (AD) | 3 | 84 | 10 | 8 | - | - |
| 4 | 85 | M | Chronic pruritus (AD) | 1 | 75 | 4 | 3 | - | - |
| 5 | 74 | F | Chronic prurigo | 3 | 72 | 9 | 6 | - | - |
| 6 | 90 | F | Chronic prurigo | 3 | 60 | 7 | 6 | - | - |
| 7 | 74 | F | Atopic dermatitis | 1 | 70 | 9 | 8 | - | - |
| 8 | 74 | F | Chronic prurigo | 3 | 70 | 8 | 5 | - | - |
| 9 | 71 | M | Chronic prurigo | 3 | 70 | 10 | 8 | - | - |
| 10 | 77 | M | Atopic dermatitis | 1 | 1 | 10 | 9 | - | - |
| 11 | 87 | F | Chronic prurigo | 3 | 62 | 8 | 2 | - | - |
| 12 | 83 | M | Chronic prurigo | 3 | 80 | 3 | 2 | - | - |
| 13 | 75 | F | Nummular dermatitis | 1 | 73 | 8 | 7 | - | - |
| 14 | 78 | M | Chronic prurigo | 3 | 72 | 7 | 2 | - | - |
| 15 | 73 | M | Grover’s disease | 1 | 72 | 7 | 5 | - | - |
| 16 | 74 | M | Atopic dermatitis | 1 | 70 | 6 | 3 | - | + |
| 17 | 73 | F | Lichen planus | 1 | 72 | 8 | 6 | - | + |
| 18 | 85 | M | Chronic prurigo | 3 | 83 | 10 | 10 | - | - |
| 19 | 92 | M | Atopic dermatitis | 1 | 91 | 5 | 3 | - | - |
| 20 | 81 | F | Chronic prurigo | 3 | 67 | 10 | 10 | - | - |
| 21 | 80 | F | Atopic dermatitis | 1 | 70 | 5 | 3 | - | - |
| 22 | 74 | F | Prurigo simplex | 1 | 74 | 7 | 5 | - | - |
| 23 | 83 | M | Asteatotic eczema | 1 | 78 | 10 | 8 | - | - |
| 24 | 72 | F | Chronic prurigo | 3 | 71 | 9 | 7 | - | - |
| 25 | 72 | M | Psoriasis | 1 | 50 | 8 | 7 | - | - |
| 26 | 82 | F | Atopic dermatitis | 1 | 80 | 10 | 5 | - | - |
| 27 | 84 | M | Drug eruption | 1 | 82 | 8 | 7 | + | - |
| 28 | 83 | F | Chronic prurigo | 3 | 56 | 7 | 5 | - | - |
| 29 | 79 | F | Chronic prurigo | 3 | 46 | 8 | 5 | - | - |
| 30 | 82 | M | Atopic dermatitis | 1 | 77 | 6 | 5 | - | - |

*Abbr.:* AD, atopic dermatitis; AI-NRS, Average Itch Numeric Rating Scale (0, no itch; 10, worst itch imaginable); AIBD, autoimmune blistering disorders; F, female; IF, immunofluorescence; IFSI, International Forum for the Study of Itch (Group 1, pruritus on primarily diseased/inflamed skin; Group 2, pruritus on unaffected skin; Group 3, pruritus with chronic secondary scratch lesions); M, male; WI-NRS, Worst Itch Numeric Rating Scale (0, no itch; 10, worst itch imaginable).

TABLE S5 Immunoserological results of patients > 70 years of age with suspected AIBD, yet without linear IgG, IgA, IgM, and C3 deposits at the cutaneous BMZ.

| **Case #** | **Indirect IF on salt-split skin (IgM)** | **Immunoblotting with BP180 NC16A (IgM)** |
| --- | --- | --- |
| 1 | Neg. | Neg. |
| 2 | Neg. | Neg. |
| 3 | Neg. | Neg. |
| 4 | Neg. | Neg. |
| 5 | Neg. | Neg. |
| 6 | Neg. | Neg. |
| 7 | Neg. | Neg. |
| 8 | Neg. | Neg. |
| 9 | Neg. | Neg. |
| 10 | Neg. | Neg. |
| 11 | Neg. | Neg. |
| 12 | Neg. | Pos. |
| 13 | Neg. | Neg. |
| 14 | Neg. | Neg. |
| 15 | Neg. | Neg. |
| 16 | Neg. | Neg. |
| 17 | Neg. | Neg. |
| 18 | Neg. | Neg. |
| 19 | Neg. | Neg. |
| 20 | Neg. | Neg. |
| 21 | Neg. | Neg. |
| 22 | Neg. | Neg. |
| 23 | Neg. | Neg. |
| 24 | Neg. | Pos. |
| 25 | Neg. | Neg. |
| 26 | Neg. | Neg. |
| 27 | Neg. | Neg. |
| 28 | Neg. | Neg. |
| 29 | Neg. | Neg. |
| 30 | Neg. | Neg. |
| 31 | Neg. | N.d. |
| 32 | Neg. | N.d. |
| 33 | Pos. | Neg. |
| 34 | Neg. | N.d. |
| 35 | Neg. | N.d. |
| 36 | Pos. | Neg. |
| 37 | Neg. | N.d. |
| 38 | Neg. | N.d. |
| 39 | Neg. | N.d. |
| 40 | Neg. | N.d. |
| 41 | Neg. | N.d. |
| 42 | Neg. | N.d. |
| 43 | Neg. | N.d. |
| 44 | Neg. | N.d. |
| 45 | Neg. | N.d. |
| 46 | Neg. | N.d. |
| 47 | Neg. | N.d. |
| 48 | Neg. | N.d. |
| 49 | Neg. | N.d. |
| 50 | Pos. | Neg. |
| 51 | Neg. | N.d. |
| 52 | Neg. | N.d. |
| 53 | Neg. | N.d. |
| 54 | Neg. | N.d. |
| 55 | Neg. | N.d. |
| 56 | Neg. | N.d. |
| 57 | Neg. | N.d. |
| 58 | Neg. | N.d. |
| 59 | Neg. | N.d. |
| 60 | Neg. | N.d. |

*Abbr.:* IF, immunofluorescence; N.d., not done; Neg., negative; Pos., positive
